# Microglial nodules in early multiple sclerosis white matter are associated with degenerating axons

**DOI:** 10.1007/s00401-013-1082-0

**Published:** 2013-01-26

**Authors:** Shailender Singh, Imke Metz, Sandra Amor, Paul van der Valk, Christine Stadelmann, Wolfgang Brück

**Affiliations:** 1 Department of Neuropathology, University Medical Center, Georg-August University Göttingen, Robert-Koch-Str. 40, 37075 Göttingen, Germany; 2 Department of Pathology, VU University Medical Center, Amsterdam, The Netherlands; 3 Neuroscience and Trauma Centre, Barts and the London School of Medicine and Dentistry, Queen Mary University of London, London, UK

**Keywords:** Wallerian degeneration, Microglial nodules, Multiple sclerosis, Preactive lesions, Axonal damage, Microglia activation

## Abstract

Microglial nodules in the normal-appearing white matter have been suggested as the earliest stage(s) of multiple sclerosis (MS) lesion formation. Such nodules are characterized by an absence of leukocyte infiltration, astrogliosis or demyelination, and may develop into active demyelinating MS lesions. Although the etiology of MS is still not known, inflammation and autoimmunity are considered to be the central components of this disease. Previous studies provide evidence that Wallerian degeneration, occurring as a consequence of structural damage in MS lesions, might be responsible for observed pathological abnormalities in connected normal-appearing white matter. As innate immune cells, microglia/macrophages are the first to react to even minor pathological changes in the CNS. Biopsy tissue from 27 MS patients and autopsy and biopsy tissue from 22 normal and pathological controls were analyzed to determine the incidence of microglial nodules. We assessed MS periplaque white matter tissue from early disease stages to determine whether microglial nodules are associated with altered axons. With immunohistochemical methods, the spatial relation of the two phenomena was visualized using HLA-DR antibody for MHC II expression by activated microglia/macrophages and by applying antibodies against damaged axons, i.e., SMI32 (non-phosphorylated neurofilaments) and amyloid precursor protein as well as neuropeptide Y receptor Y1, which marks axons undergoing Wallerian degeneration. Our data demonstrate that the occurrence of microglial nodules is not specific to MS and is associated with degenerating as well as damaged axons in early MS. In addition, we show that early MS microglial nodules exhibit both pro- and antiinflammatory phenotypes.

**Electronic supplementary material:**

The online version of this article (doi:10.1007/s00401-013-1082-0) contains supplementary material, which is available to authorized users.

## Introduction

Multiple sclerosis (MS) is a chronic inflammatory CNS disease characterized by multifocal inflammation, extensive demyelination, gliosis and axonal damage. The etiology of MS is still not known, and the first cellular events in the MS brain remain to be clarified. Microglia are the resident immune cells that react to even minor pathological events in the CNS [27], and microglial activation might arguably be considered an initial pathogenetic event in MS. Inflammation and microglia/macrophage activation may cause myelin/axonal damage or, alternatively, the cells may scavenge damaged myelin/axonal debris. A cluster of activated microglia/macrophages is commonly termed a ‘microglial nodule’ without a strict definition of the cell number. Studies of post-mortem MS brain tissues have proposed that microglia nodules observed in the so-called normal-appearing white matter (NAWM) may represent the earliest stage(s) in MS lesion development and were therefore called (p)reactive lesions [13, 46, 52, 55]. Such nodules are identified in the absence of leukocyte infiltration, astrogliosis or demyelination [53, 55]. Microglial nodules are a well-known phenomenon in viral encephalitis, in which perineuronal aggregation of activated microglia/macrophages indicative of neuronal phagocytosis is often observed. Furthermore, phagocytosis of neuroaxonal debris by activated microglia/macrophages is in agreement with prior observations in MS [25]. In fact, activated microglia/macrophages also release a wide range of cytotoxins, free radicals, neurotrophic factors and immunomodulatory molecules, which may reflect immunoregulatory rather than just proinflammatory activity [8, 23]. Two extremes of these activation states are classically activated (M1) and alternatively activated (M2) macrophages.

Axonal transection within the lesion, causing anterograde degeneration of the distal part of the axon—also known as Wallerian degeneration—might occur diffusely in the tissue surrounding the lesion, inducing microglial/macrophage reaction to absorb axonal and myelin fragments in non-demyelinated MS white matter. Moreover, results of several neuropathological and neuroradiological studies confirmed Wallerian degeneration in early MS NAWM [10, 11, 18, 21, 48]. Based on MS periplaque white matter (PPWM) biopsy tissue, a recent study using an antibody against the neuropeptide Y receptor Y1 (NPY-Y1R) also showed widespread Wallerian degeneration [19]. The antigen detected by the anti-NPY-Y1R antibody remains to be determined. It is however known that peripheral nerve injury induces increased NPY expression in dorsal root ganglion neurons in rodents, and receptor Y1 is upregulated in neurons [12, 57, 58, 61]. Thus, it is possible that NPY-Y1R plays a role in promoting survival during degenerative processes.

The main aim of our study was to investigate white matter biopsy tissue from early stage MS patients in order to determine whether axonal alterations are present in microglial nodules. To do so, we used anti-HLA-DR (an antigen belonging to human leukocyte-associated antigens class II) antibody for activated microglia/macrophages and three different immunohistochemical markers for damaged axons, i.e., SMI32, which reacts to damaged axons with non-phosphorylated neurofilaments; amyloid precursor protein (APP) as a marker for transport disturbance in acutely damaged axons; and NPY-Y1R for axons undergoing Wallerian degeneration. Subsequently, we characterized the immune phenotype of MS microglial nodules using M1 and M2 macrophage markers. Based on the literature, we selected antibodies directed against CD40 and inducible nitric oxide synthase (iNOS) as M1 markers and mannose receptor and CD163 as M2 markers [3, 5, 23, 26, 62]. A better understanding of pathological processes in non-demyelinated white matter in early MS may enable prevention of further damage in the CNS through rational therapeutic strategies.

## Materials and methods

### Patients

We investigated biopsy tissue from 27 patients who had been diagnosed with inflammatory demyelination of the CNS consistent with MS. A total of 44 tissue blocks, which included non-demyelinated white matter regions, were used for the study. The biopsies were performed in different neurosurgery centers for various diagnostic reasons, for example, to exclude neoplastic or infectious diseases. Informed consent had been obtained from each patient. None of the study authors was involved in decision making with respect to biopsy. However, histology showed inflammatory demyelinating lesions typical for MS. Specimens were sent to the Department of Neuropathology in Göttingen, Germany, for a second opinion. Patients received the diagnosis of MS according to the McDonald’s or Poser’s criteria [36, 43]. Of the 27 patients, 8 underwent comprehensive clinical follow-up: 6 patients (MS nos. 3, 4, 7, 13, 19, 25) had a relapsing-remitting course, MS no. 11 showed all the characteristics of primary progressive MS [51], and MS no. 27 entered a secondary progressive disease course years after biopsy. All the other 19 patients were diagnosed with clinically isolated syndrome suggestive of MS. Patient characteristics are summarized in Supplement 1.

We also analyzed archival paraffin-embedded brain tissue obtained from seven brain infarct (four autopsy and three biopsy) cases, three traumatic brain injury (TBI) autopsies, and seven biopsies from patients who underwent surgery for temporal lobe epilepsy. The control group consisted of autopsy CNS tissue from five patients who died suddenly because of non-neurological disorders. No neurological abnormalities were found in any routine autopsy control cases; in particular, there were no signs of inflammation, hypoxia or neuronal damage. The interval between death and autopsy ranged from 24 to 48 h (median = 24 h) in infarct, 10 to 24 h (median = 22 h) in TBI and 8 to 120 h (median = 72 h) in control cases.

The study was carried out according to the national ethics guidelines and legal regulations regarding the use of archival post-mortem material. All patients and controls, or their next of kin, had given informed consent for autopsy.

### Histopathology

**Fig. 1:**
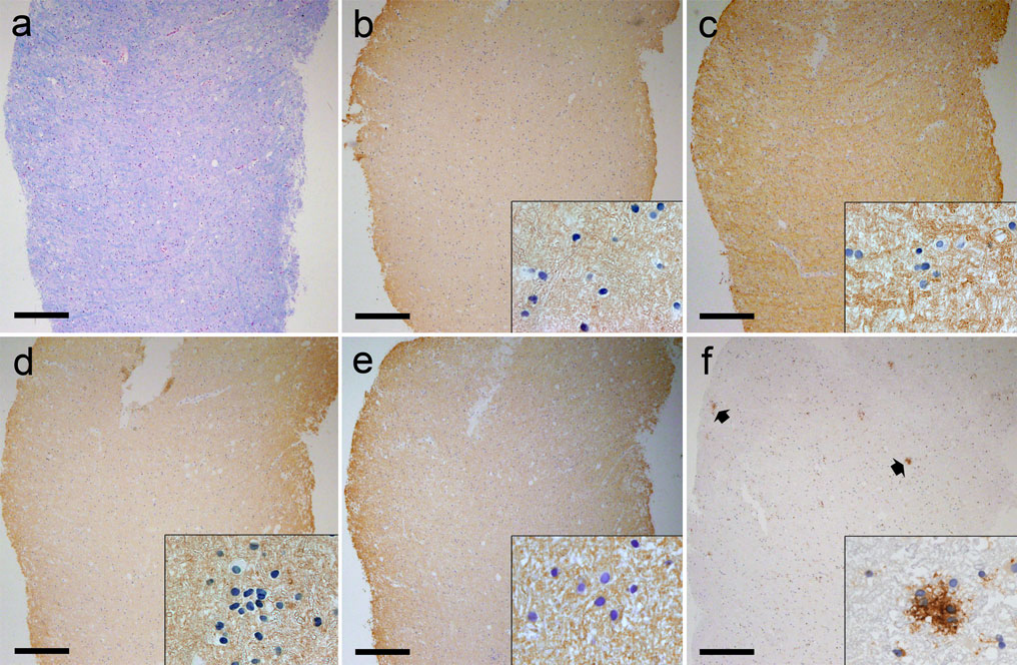
Microglial nodules are found in the normally myelinated periplaque white matter in MS biopsy tissue. Staining for LFB/PAS (**a**) and immunohistochemistry for PLP (**b**), MBP (**c**), MOG (**d**) and MAG (**e**) on sequential tissue sections showed normal myelin (MS no. 7). Activated microglia/macrophages and circumscribed nodules (*arrows*) that express HLA-DR are shown in (**f**). *Insets* in (**b**, **c**, **d**, **e**, **f**) show higher magnification of the respective tissue immunostaining. *Scale bars* = (**a**–**f**) 250 μm

Specimens were fixed in 4 % paraformaldehyde and embedded in paraffin. Sections 4 μm thick were stained with hematoxylin and eosin (HE), luxol fast blue (LFB)/periodic acid-Schiff (PAS) or Bielschowsky’s silver impregnation. Immunohistochemical staining was performed with a biotin–avidin or an alkaline phosphatase/anti-alkaline phosphatase technique. The primary antibodies used for diagnostic purposes were: anti-myelin basic protein (anti-MBP, rabbit polyclonal, DakoCytomation, Glostrup, Denmark), anti-proteolipid protein (anti-PLP, mouse monoclonal, Biozol, Eching, Germany), anti-myelin oligodendrocyte glycoprotein (anti-MOG, rat monoclonal, kindly provided by Prof. Linington, University of Glasgow, UK), anti-myelin-associated glycoprotein (anti-MAG, rabbit polyclonal, kindly provided by Prof. Schwab, University of Zürich, Switzerland), anti-cyclic nucleotide phosphodiesterase (anti-CNPase, mouse monoclonal, Covance Inc., Princeton, NJ, USA), KiM1P (macrophages, mouse monoclonal, kindly provided by Prof. Radzun, University of Göttingen, Germany) and anti-MRP14 (activated macrophages, mouse monoclonal, BMA Biomedicals, Augst, Switzerland), anti-CD3 (T cells, rat monoclonal, Serotec, UK), anti-CD8 (cytotoxic T cells, rabbit polyclonal, Dako, Denmark), anti-glial fibrillary acidic protein (anti-GFAP, rabbit polyclonal, Dako, Denmark), anti-IgG (plasma cells, rabbit polyclonal, Dako, Denmark) and anti-complement C9neo antigen (anti-C9 neo, rabbit polyclonal, kindly provided by Prof. Morgan, University of Cardiff, UK). In order to detect damaged axons, we used anti-non-phosphorylated neurofilaments (SMI32, mouse monoclonal, Sternberger MD, USA) and anti-amyloid precursor protein (anti-APP, mouse monoclonal, Millipore, MA, USA). Wallerian degeneration was visualized using anti-neuropeptide-Y1 receptor (anti-NPY-Y1R, rabbit polyclonal, Acris Antibodies, Hiddenhausen, Germany). PPWM in all the 44 tissue blocks demonstrated normal myelin staining with the above-mentioned myelin markers. The occurrence of microglial nodules was assessed by anti-HLA-DR (MHC II, mouse monoclonal, clone LN3, eBioscience, USA) (Fig. 1). All the detected microglial nodules and their surroundings were thoroughly investigated to verify the absence of leukocyte infiltration and astrogliosis on sequential tissue sections (data not shown). Immunohistochemistry was performed on MS biopsy tissue to detect expression of pro- and antiinflammatory markers in microglial nodules using antibodies against CD40 (anti-CD40, rabbit polyclonal, Acris Antibodies, Hiddenhausen, Germany), inducible nitric oxide synthase (anti-iNOS, rabbit polyclonal, Millipore, MA, USA), mannose receptor (anti-mannan binding protein, mouse monoclonal, Serotec, UK) and CD163 (anti-CD163, mouse monoclonal, Novo Laboratories, UK). Omission of primary antibody served as a control staining.

The antibody against NPY-Y1R used in this study stains degenerating nerve fibers and has been investigated in epilepsy, brain infarct and MS tissue previously [19, 42]. However, the antigen detected by this antibody in degenerating axons remains unknown. In the present study, we have further analyzed Wallerian degeneration in the axial dimension of MS PPWM biopsy tissue sections using a Z-stack module of confocal laser scanning microscopy. For fluorescent staining, sections containing microglial nodules were double-stained for HLA-DR and NPY-Y1R. Cy3-labeled goat anti-mouse and Alexa 488-labeled goat anti-rabbit were used as secondary antibodies.

### Classification of multiple sclerosis lesions

All biopsy specimens fulfilled the generally accepted criteria for the pathological diagnosis of MS, showing an inflammatory demyelinating lesion [31, 44]. Lesions were classified according to their demyelinating activity as described in detail earlier [9]. Early active lesions were infiltrated by numerous macrophages that are immunoreactive with major and minor myelin proteins (MBP, PLP, MAG, MOG, CNP). In late active lesion areas, degradation of myelin proteins was more advanced, and macrophages contained only MBP- (and PLP-) but not MOG- (or MAG-) positive myelin debris. Inactive lesions were completely demyelinated, and macrophages no longer contained myelin protein-positive degradation products within their cytoplasm. In early remyelinating plaques, thin, irregularly formed myelin sheaths were seen as well as a pronounced infiltration by macrophages/microglial cells and T cells. Remyelination was more advanced in late remyelinating lesions, and only a few inflammatory cells could be found.

### Image analysis and experimental details

Tissue sections were analyzed using an Olympus BX51 fluorescence microscope equipped with a DP71 CCD camera (Olympus Optical Co, Ltd., Hamburg, Germany) and a Zeiss Cell Observer microscope with an AxioCam ICc 3 CCD camera (Carl Zeiss MicroImaging, Ltd., Göttingen, Germany) or by confocal laser scanning microscopy with a Fluoview 1000 Olympus microscope. All the images were prepared in Adobe Photoshop CS4, version 11.0.2. The numbers of cells constituting microglial nodules were counted as visualized on HLA-DR stained sections using light microscopy. For measuring the distances on tissue section, the analySIS^®^ image processing software was used. Evaluation of damaged axons associated with microglial nodules was done by measuring the distance between the nearest APP^**+**^/SMI32^**+**^ axonal structure and nodule-forming nuclei. Three-dimensional reconstruction from confocal Z-stack images was performed using Imaris Bitplane scientific software, version 7.5.

### Statistical analysis

Differences in the number of microglial nodules against lesion activity were statistically analyzed by Mann-Whitney *U* test. Statistical analysis for the group difference between microglial nodules containing APP-positive axonal profiles and microglial nodules lacking APP-positive axonal profiles was performed using non-parametric *t* tests. Statistical significance was defined as *p* < 0.05.

## Results

### Incidence of microglia nodules in white matter from patients with early stage MS

To screen for microglial nodules, we examined PPWM tissue from 27 biopsied MS patients with a median interval from symptom onset to biopsy of 22.5 days (range, 3 days–11 years). Identification of microglial nodules in the tissue blocks containing PPWM was based on sequential immunohistochemical staining of tissue sections for MHC II (HLA-DR) and myelin markers, i.e., LFB/PAS, PLP, MBP, MOG and MAG (Fig. 1). Microglial nodules (HLA-DR^+^) showing variable morphologies, composed of four or more cells, were observed in 16 patients (59.3 %) and 20 blocks (45.5 %). The median number of cells constituting microglial nodules was 9 cells per nodule and ranged from 4 to 18 cells per nodule. The median time to biopsy was 21 days (range, 3–44 days) for the patients containing microglial nodule(s). We observed that the microglial nodules in PPWM occurred more frequently in patients with early active and late active lesions compared to patients with inactive lesions (Table 1). However, the quantitative determination of microglial nodules against lesion activity revealed that microglial nodules in PPWM occur irrespective of the plaque activity. There was no significant difference in the number of nodules associated with early active as compared to late and inactive lesions (*p* = 0.11); similarly, no significant difference was found in the number of nodules associated with active demyelinating (i.e., both early and late active) lesions compared to inactive lesions (*p* = 0.45). Nonetheless, we cannot exclude the possibility that the studied PPWM region containing microglial nodules could also be in the proximity of other lesions not included in the studied tissue specimens. In addition, the shortest possible distances between microglial nodules and the nearest blood vessel present in the tissue sections were measured; the median distance was calculated at 541.76 μm (range 81.47–1,554.75). We further investigated nodule-forming microglia/macrophages using the anti-MRP14 antibody. Monocytes express MRP14 early in their activation phase and during tissue invasion until their terminal differentiation to macrophages, but none of the microglial nodules or the surrounding microglia/macrophage cells were found to be MRP14-positive on sequentially stained tissue sections (data not shown) [9].

**Table 1:** Microglial nodule occurrence versus lesion activity

MS no.	No. of microglial nodule(s)	Lesion activity	Source
1	3	Inactive	Left parietal
2	3	Inactive	Right frontal
3	2	Late active	Right occipital
4	7	Early active	Corpus callosum
5	1	Late active	Right frontal
6	9	Inactive	Left occipital
7	3	Early active	Right occipital
8	1	Early active	Right frontal
9	4	Inactive	Right frontal
10	1	Early active	Left temporal
11	1	Late active	Right parietal
12	4	Inactive	Left parietal
13	5	Inactive	Left temporal
14	1	Late active	Left frontal
15	2	Early active	Left occipital
16	1	Inactive	Right frontal
17	Not present	Inactive	Right frontal
18	Not present	Inactive	Occipital
19	Not present	Inactive	Right frontal
20	Not present	Inactive	Right temporal
21	Not present	Inactive	Right frontal
22	Not present	Inactive	Right occipital
23	Not present	Inactive	Left temporal
24	Not present	Late active	Left frontal
25	Not present	Inactive	Left frontal
26	Not present	Late active	Right frontal
27	Not present	Inactive	Left frontal

### Microglial nodules are associated with damaged axons in early MS

**Fig. 2:**
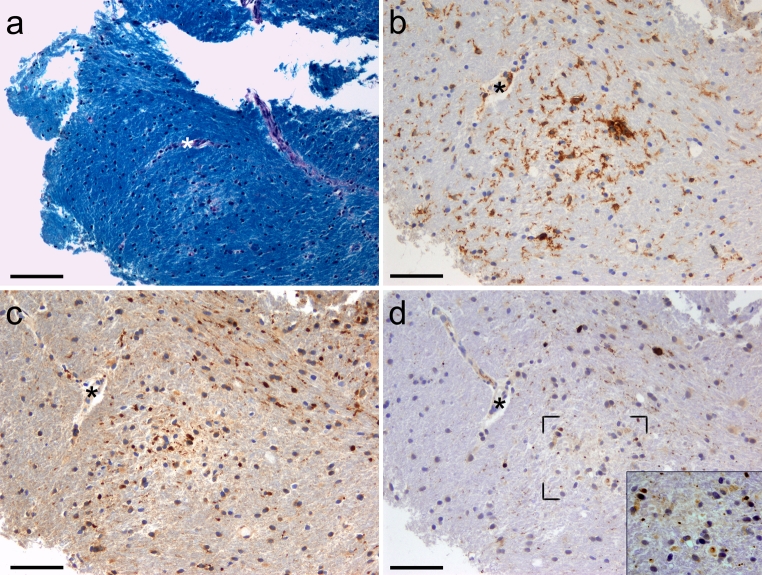
Microglial nodules are associated with underlying axonal pathology in early multiple sclerosis. LFB/PAS stain and HLA-DR immunohistochemistry were used to identify microglial nodules in PPWM (**a**, **b**). Sequential tissue sections (**a**, **b**, **c**, **d**) were matched using a blood vessel (*asterisk*) (MS no. 5). The region of interest exhibits intact myelin (**a**). Microglial nodule representing cluster of activated HLA-DR expressing microglia/macrophages localized in normally myelinated PPWM tissue (**b**). Sequential section identified injured/damaged axons associated with microglial nodules (**c**, **d**). APP^+^, acutely damaged axons are detected in close association with the microglial nodule (**c**). SMI32^+^, axonal ovoids occur in the same region (**d** and *inset*). *Inset* in (**d**) shows higher magnification of the marked region. *Scale bars* = (**a**) 100 μm; (**b**–**d**) 50 μm

Sequential tissue sections of PPWM were immunostained for HLA-DR and axonal damage markers, i.e., APP and SMI32. Intra-axonal APP accumulation representing disturbed axonal transport was observed along with terminal ovoids resembling small bulbs, whereas SMI32 stained morphologically intact axons as well as axons with alternating constrictions or single swellings as dot-like ovoids. Analysis of sequential tissue sections revealed that microglial nodules and surrounding activated microglia/macrophages were associated with damaged axons (Fig. 2). All 48 microglial nodules studied were found to be associated with SMI32^**+**^ axons, while APP^**+**^ axons and ovoids were detected in more than half of the studied nodules (Table 2). APP^**+**^/SMI32^**+**^ axonal structures were detected at a median distance of 4.22 μm (range 2.97–5.68) from the nuclei of microglial nodules, which were considered to be associated with damaged axons. A non-parametric *t* test was performed to evaluate occurrence of microglial nodules with APP^+^ axonal structures; there was no significant difference in the number of APP-positive and APP-negative nodules (*p* = 0.37).

**Table 2:** Microglial nodules examined for damaged axons

Total number of nodules examined: 48
Nodules associated with APP^+^ axons (%): 29 out of 48 (60.42 %)
Nodules associated with SMI32^+^ axons (%): 48 out of 48 (100 %)
Nodules associated with NPY-Y1R^+^ axons (%): 27 out of 27 (100 %)

### Simultaneous detection of microglial nodules and axons undergoing Wallerian degeneration

**Fig. 3:**
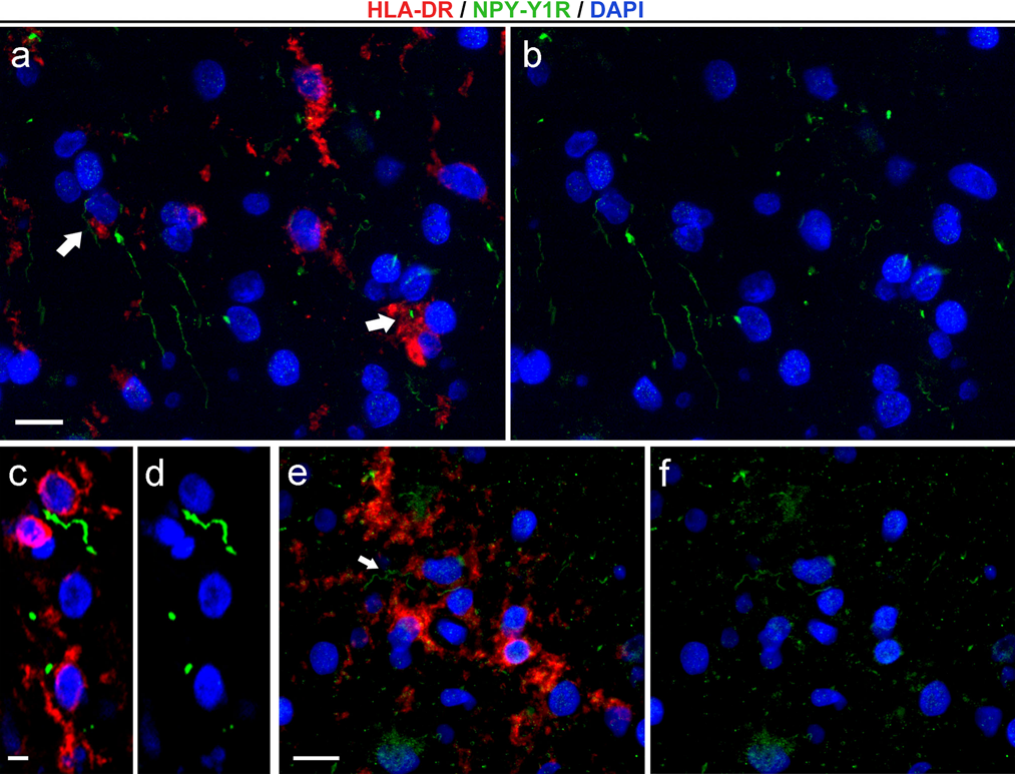
Axons undergoing Wallerian degeneration in close spatial association with activated microglia/macrophages in MS PPWM. NPY-Y1R^+^ axons (*green*) undergoing Wallerian degeneration apposed to HLA-DR^+^ microglia/macrophages (*red*) with an activated morphology (*arrows*, **a**–**b**) (MS no. 14). NPY-Y1R^+^ axons were frequently surrounded by activated microglia/macrophages throughout the PPWM (**c**–**d**) (MS no. 1). Activated microglia/macrophage cells were visualized clustering along the length of the NPY-Y1R^+^ axonal segment (*arrow*, **e**–**f**) (MS no. 4). Stainings are merged with DAPI, which stains the nuclei (**a**–**f**, *blue*). *Scale bars* = (**a**–**b**) 25 μm; (**c**–**d**) 10 μm; (**e**–**f**) 20 μm

**Fig. 4:**
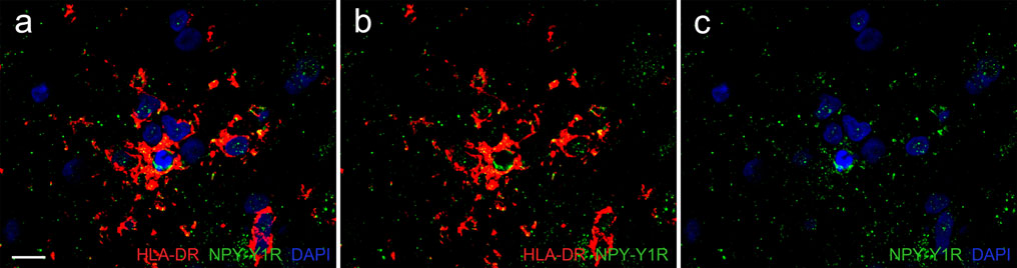
Degenerating axons undergoing Wallerian degeneration associated with microglial nodule in MS. Fluorescent immunohistochemical staining for HLA-DR (*red*) counterstained with DAPI (*blue*) shows a microglial nodule consisting of several microglial/macrophage cells (MS no. 11). The association of NPY-Y1R^+^ fragments (*green*) with the microglial nodule suggests Wallerian degeneration induced formation of microglial nodule. *Scale bar* = 30 μm

**Fig. 5:**
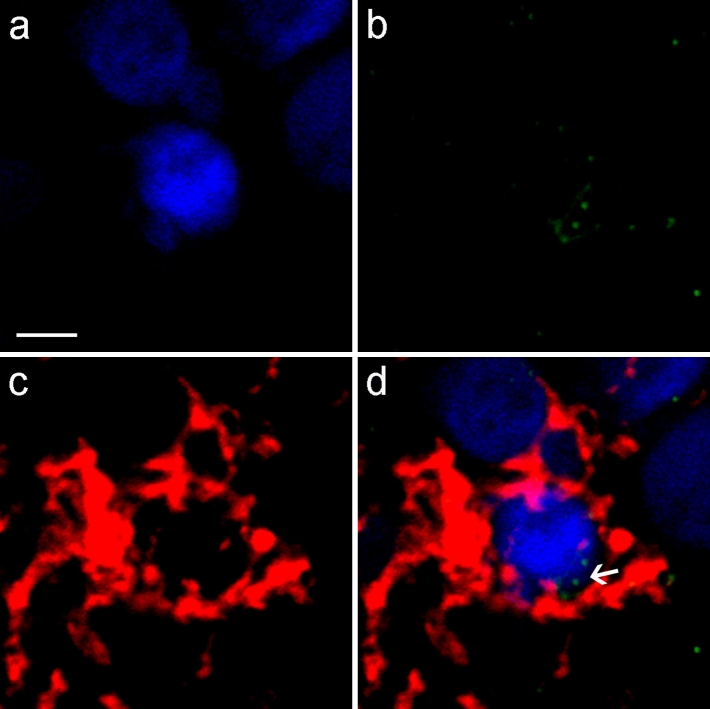
Intracellular NPY-Y1R^+^ axonal fragments in a HLA-DR^+^ nodule-forming microglial/macrophage cell. Nuclei of nodule-forming microglia/macrophage cells (*blue*, **a**), NPY-Y1R^+^ particles (*green*, **b**) present within the microglial/macrophage cytoplasm (*red*, **c**). The merge is shown in (**d**). The presence of intracellular NPY-Y1R^+^ fragments (*arrow*) in nodule-forming HLA-DR^+^ cell indicates engulfment of axonal debris by activated microglia/macrophages. *Scale bar* = 15 μm

To investigate the relationship between microglial nodules and the process of Wallerian degeneration, double-labeled (i.e., HLA-DR and NPY-Y1R antibodies) immunostained sections from nine cases (MS nos. 1, 3, 4, 5, 7, 9, 11, 13, 14) were visualized using the confocal laser-scanning microscopy technique. All the 27 HLA-DR^+^ nodules from examined cases were found to be associated with NPY-Y1R^+^ structures (Table 2). The nodules were closely apposed to and sometimes clustering around degenerating axons as well as the activated, nodule-free microglia/macrophage cells (Fig. 3). A direct spatial association was observed between microglial nodules and axons undergoing Wallerian degeneration (Fig. 4). The presence of NPY-Y1R^+^ fragments in nodule-forming HLA-DR^+^ microglia/macrophage cells clearly underlines the functional relationship between degenerating axons and microglial/macrophage reaction (Fig. 5). This association is further visualized in Supplement 2. Quite intriguingly, not all NPY-Y1R^+^ profiles were surrounded by HLA-DR^+^ cells, which might indicate axonal degeneration is primary to microglia/macrophage activation of the type seen in microglial nodules [47].

### Microglial nodules are not specific to MS

**Fig. 6:**
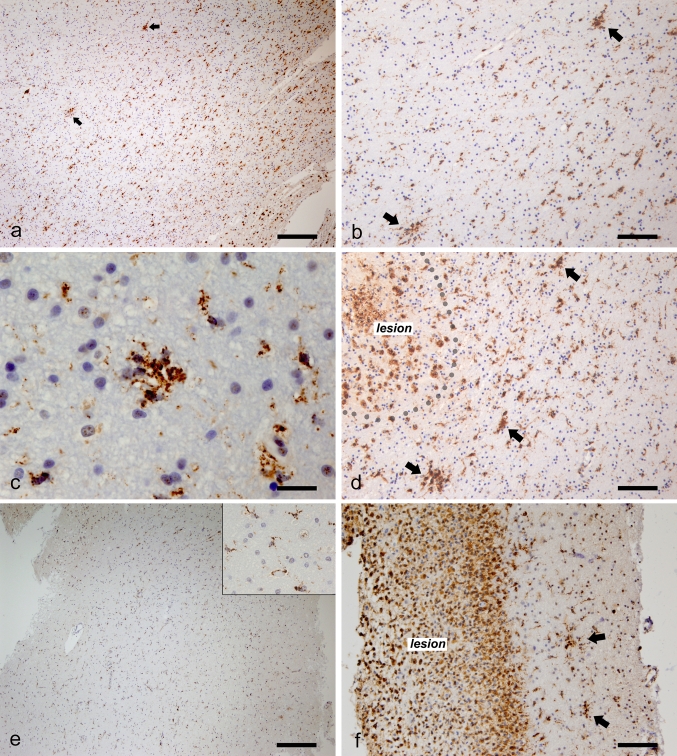
Identification of microglial nodules in OND and MS. Microglia/macrophage activation (HLA-DR^+^) throughout the perilesional white matter in the autopsy tissue from an infarct case (**a**). **b** Represents a magnification of the region containing nodules in (**a**). Microglial nodule in the white matter biopsy tissue from a patient with cerebral infarction (**c**). White matter region surrounding contusional lesion in an autopsy case (**d**). Disperse microglial/macrophage activation in biopsied epilepsy white matter (**e**, *inset*). Nodules present adjacent to the biopsied MS lesion in MS no. 3 (**f**). Immunohistochemistry for HLA-DR: (**a**–**f**). *Arrows*: nodules. *Scale bars* = (**a**,** e**) 250 μm; (**b**,** d**,** f**) 100 μm; (**c**) 25 μm

To gain more insight into the prevalence of microglial nodules, we systematically screened autopsy and biopsy tissue from other neurological diseases (OND). Further details of OND, i.e., infarct, TBI, epilepsy and control cases, are summarized in Table 3. H&E, LFB/PAS and HLA-DR staining was used to identify the nodules in normal white matter tissue. The occurrence of microglial nodules was observed in two TBI and all the seven infarct patients, whereas nodules were absent in epilepsy and non-neurological control patients. TBI and infarct lesions were characterized by strong macrophage infiltration and profound axonal loss and axonal spheroids; we found abundant microglial nodules in the affected adjacent normal white matter (Fig. 6a–d). In patients who underwent surgery because of epilepsy, neuropathological examination revealed no significant abnormalities except for mild astrogliosis in two cases. Only a modest level of microglia/macrophage activation was present in the white matter; activated microglia/macrophages were almost evenly dispersed, and no microglial/macrophage clustering was observed in any of the epilepsy patients (Fig. 6e) [7].

**Table 3:** Characteristics of OND and control cases

	No. of cases	Median age (years)	F/M ratio	Total no. of samples studied^a^	No. of patients with microglial nodule(s)
Infarct					
Autopsy	4	59.5 (range, 43–86)	1/3	4	4
Biopsy	3	62 (range, 49–72)	0/3	5	3
TBI					
Autopsy	3	44 (range, 18–47)	0/3	3	2
Epilepsy					
Biopsy	7	39 (range, 28–48)	3/4	11	–

Normal controls	Underlying disease	Cause of death	Age (years)	Sex	No. of samples studied^a^	Microglial nodule(s)
1	None	Multi-organ failure	51	M	2	–
2	None	Aortic dissection	71	M	3	–
3	Unknown	Acute cardiac failure	64	F	2	–
4	CAD	Acute cardiac failure	58	F	1	–
5	Unknown	Acute cardiac failure	37	F	1	–

*CAD* coronary artery disease

– no nodules detected in the any of the samples

^a^Only white matter region(s) present in each sample were studied

### Expression of M1 and M2 markers in activated microglia/macrophages and microglial nodules in MS

**Fig. 7:**
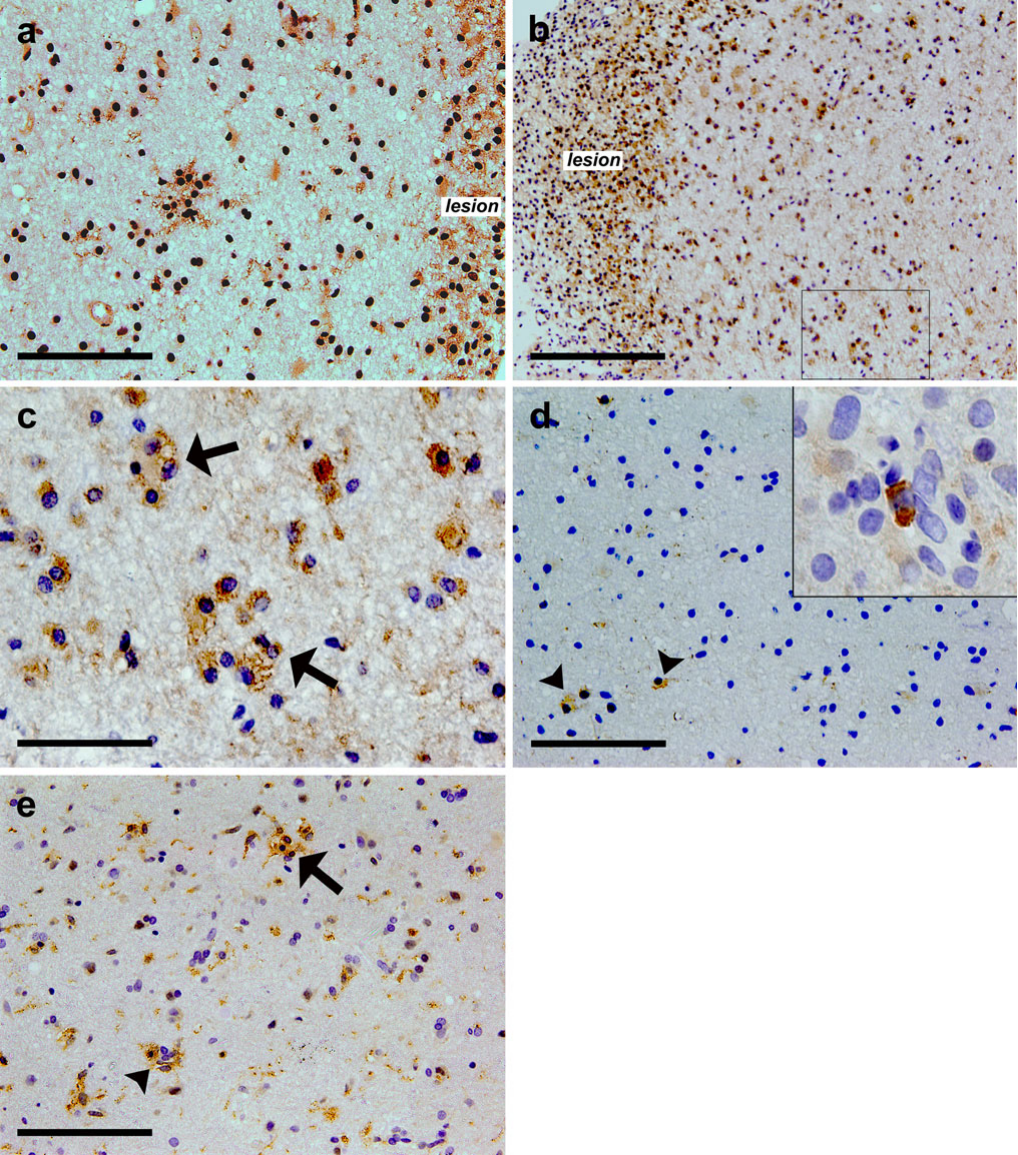
Microglial nodules in MS display both classically (M1) and alternatively (M2) activated macrophage markers. CD40 was expressed in cells associated with microglial nodules present in PPWM (**a**) (MS no. 13). iNOS was present in phagocytes in demyelinating MS lesions and very frequently found to be expressed in the clusters forming a small foci of microglial/macrophage cells (*arrows*) in PPWM (**b**, **c**) (MS no. 6). **c** Represents a higher magnification of the PPWM region marked with black box in (**b**). M2 marker mannose receptor was present perivascularly (*inset* of **d**) and was only rarely expressed on parenchymal cells (*arrowheads*) in PPWM (**d**) (MS no. 13). CD163 was detected in activated microglia/macrophages and microglial nodules (*arrow*) as well as in perivascular spaces (*arrowhead*) (**d**) (MS no. 8). *Scale bars* = (**a**,** d**,** e**) 100 μm; (**b**) 200 μm; (**c**) 50 μm

To determine the immune phenotype of microglial nodules in MS, we analyzed the expression of pro- and antiinflammatory markers characteristic for M1 and M2 macrophages. Microglia/macrophages associated with microglial nodules express both pro- and antiinflammatory markers (Fig. 7). Evaluation was performed on five cases (MS nos. 3, 6, 8, 13, 15) using antibodies against various surface proteins involved in antigen presentation and recognition, i.e., CD40, iNOS, mannose receptor and CD163. CD40 and iNOS, proinflammatory molecules, were highly expressed throughout the MS PPWM and frequently observed in microglial nodules and activated microglia/macrophage clusters. CD163, a marker for antiinflammatory M2 macrophages, primarily stained perivascular macrophages but was also present in parenchymal PPWM microglia/macrophages in two cases (MS nos. 8 and 13). In contrast, the immunoreactivity of mannose receptor was mostly limited to perivascular macrophages and was only occasionally positive for parenchymal microglia/macrophages in PPWM regions.

## Discussion

Our present study shows that microglial nodules are associated with damaged axons in early MS PPWM. Intra-axonal APP accumulation and changes of neurofilament phosphorylation served as a markers for axonal damage. Axons undergoing Wallerian degeneration showed granular degeneration and were detected using the anti-NPY-Y1R antibody. Here, we sought to clarify that microglial nodule formation is reactive in early MS white matter.

Over the last 2 decades, microglial nodules have been frequently noted in MS pathology. Prineas and colleagues [1, 45] detected deposits of activated complement (C3d) in elongated microglial nodules in MS PPWM, which suggests complement-mediated clustering of activated microglia/macrophages. Another study illustrated the role of oligodendrocyte stress in microglial nodules, although the ultimate cause of this abnormality remains unclear [55]. However, oligodendrocytes exist in close relationship with axons; thus, stressed axons might very well serve as an initiating source. These data together with our findings may explain that Wallerian degeneration precedes oligodendrocyte stress and complement activation in the course of microglial nodule formation.

Microglial nodules were observed irrespective of disease duration in MS NAWM [46, 54]. One particular report studied 52 post-mortem MS cases and illustrated that the incidence of microglial nodules decreases with the increase in disease duration [46]. Given that most of the microglial/macrophage clusters in MS were found to be present in close vicinity to demyelinated plaques [46, 52, 54], we considered a cohort of biopsied patients with median disease duration of 22.5 days ideal for studying microglial nodules close to disease onset. Our data, in contrast to prior studies based on autopsy material from chronic MS patients, found altered/damaged axons associated with microglial nodules. Previous neuropathological findings addressing structural damage in MS lesions support the idea that the occurrence of microglial nodules in MS is reactive [6, 21, 29]. Stainings in the PPWM-containing nodules appeared normal for all the given myelin markers, a finding that further rules out myelin abnormalities as the primary trigger [46, 54]. This in turn also supports that myelin breakdown/clearance is a delayed and slow process in CNS Wallerian degeneration [47, 56]. Neuroradiological observations showing an association of regional lesion load and axonal density in related NAWM suggest that the observed abnormalities in non-demyelinated white matter may have at least partly developed as a result of axonal transections within lesions [14, 20, 37, 59]. In line with this, the presence of NPY-Y1R^+^ axons within the microglial nodules indicates that these nodules may have formed as outcome sequelae of Wallerian degeneration in PPWM caused by lesional pathology.

Indeed, microglial nodules are regularly seen in brain trauma [22], and in this study, supporting the earlier finding, we observed microglial nodules in the perilesional white matter of brain infarcts and TBI lesions but not in epilepsy and control cases. Also, in our previous study, we demonstrated high numbers of NPY-Y1R^+^ degenerating axons in infarct tissue while axonal degeneration was virtually absent in epilepsy patients. This underlines that the microglial nodules in CNS white matter tend to occur in overt axonal degenerative conditions and therefore may develop in various brain disorders. Microglial nodule formation induced by anterograde axonal degeneration was also observed in mice, which further supports the notion that the event of nodule formation is driven by axonal degeneration in CNS white matter [16]. Recent magnetic resonance 7T T_2_* imaging studies revealed that MS white matter lesions are perivenous [17, 50]. Besides MS, acute disseminated encephalomyelitis (ADEM) and even experimental autoimmune encephalomyelitis (EAE) lesions are known to develop as a rule around a central vein(s) [28, 32, 60]. On this basis, it seems uncertain that the microglial nodules in MS can expand to form a new, full-blown demyelinating lesion involving a pathogenesis that excludes blood-brain barrier alterations. However, De Groot et al. [13] also described clusters of microglial/macrophage cells contiguous with blood vessels containing CD45-positive lymphocytes as (p)reactive; in this context, we did not find any microglial nodule directly linked with blood vessels in the present study [54].

Our results also suggest that mechanisms other than Wallerian degeneration may contribute to axonal damage associated with microglial nodules in MS. The association of SMI32^+^ axons and frequent accumulation of APP^+^ axonal end bulbs with microglial nodules and the surrounding activated microglia/macrophages in MS white matter supports the hypothesis that microglial inflammation can have detrimental effects and mediate axonal damage [24, 30, 38, 41]. Previous reports demonstrated that inflammatory mediators secreted from activated microglia/macrophages such as matrix metalloproteinases, proinflammatory cytokines, nitric oxide and reactive oxygen species may cause disruption of axoplasmic transport and axon integrity, eventually leading to axotomy [4, 15, 39, 40, 49]. Maeda and Sobel [34] detected matrix metalloproteinases activity, while van Horssen et al. [54] observed NADPH oxidase, TNF-alpha and IL-10 expression in MS microglial nodules. Interestingly, we observed strong CD40, iNOS and CD163 expression in these foci. This may explain the inconsistency in the occurrence of APP^+^ axonal profiles in microglial nodules, i.e., that antiinflammatory mechanisms could possibly restrain the damage caused in response to focal microglia/macrophage activation. Despite this, we do not have a complete explanation for these phenotypes. However, the time factor following the trigger could play a decisive role, as the initial proinflammatory niche of activated microglia/macrophages appears to shift towards the antiinflammatory [8]. Yet, all the nodules studied contained SMI32^+^ profiles, which further suggests that the axons in PPWM/NAWM are more susceptible to altered patterns of phosphorylation of neurofilament proteins as compared to intra-axonal APP accumulation [19]. Another possible cause for APP^+^ and SMI32^+^ axonal structures detected in PPWM could be a direct anti-axonal immune response, e.g., anti-neurofascin antibodies, which selectively target neurofascin at the nodes of Ranvier in animal models and may subsequently lead to local microglia/macrophage activation [35].

In conclusion, the functional relationship between NPY-Y1R^+^ axons and microglial nodules observed in this study demonstrate that the process of axonal degeneration may cause microglial nodule formation, at least in early MS PPWM. In light of this, our data do not appear to concur with the notion that the pathogenesis of (p)reactive lesions is similar to other analogous MS lesions, such as pattern III lesions and newly forming lesions, both of which are clearly described in terms of oligodendrogliopathy and myelin loss [2, 33]. Considering the fact that pathology can provide only a snapshot of an on-going course of action in the evolving disease, immune-regulatory phenotyping of such nodules in MS may reveal its overall significance in the disease.

## Electronic supplementary material

Below is the link to the electronic supplementary material.

Supplementary material 1 (DOCX 15 kb)

3D image reconstruction showing NPY-Y1R^+^ particles are present within activated microglia/macrophages. Phagocytosed NPY-Y1R^+^ profiles (green) are present in close proximity to the nucleus (blue, counterstained with DAPI) of activated HLA-DR^+^ microglia/macrophages (red); this demonstrates the microglial/macrophage reaction to Wallerian degeneration in early MS non-demyelinated white matter (AVI 12414 kb)
