# Supplementary material for: Microglial nodules in early multiple sclerosis white matter are associated with degenerating axons
Source: Acta Neuropathol. 2013 Jan 26;125(4):595–608. doi: 10.1007/s00401-013-1082-0 (PMC3611040; doi:10.1007/s00401-013-1082-0)
Supplement: Supplementary file 1 — Supplementary material 1 (DOCX 15 kb) [file 401_2013_1082_MOESM1_ESM.docx]

**Supplementary material**

Supplement 1: Characteristics of MS patients

| **MS no.** | **Age (years)** | **Gender** | **Disease duration (days)^a^** | **No. of tissue blocks^b^** | **APP^+^ microglial nodule(s)** |
| --- | --- | --- | --- | --- | --- |
| 1 | 35 | F | 3 | 2 | - |
| 2 | 29 | M | n.a. | 2 | + |
| 3 | 37 | F | 44 | 1 | + |
| 4 | 31 | F | 39 | 2 | - |
| 5 | 39 | M | 21 | 1 | + |
| 6 | 30 | F | 44 | 3 | + |
| 7 | 24 | F | 36 | 2 | - |
| 8 | 38 | M | 21 | 1 | - |
| 9 | 34 | M | 10 | 1 | + |
| 10 | 26 | M | 4 | 1 | - |
| 11 | 19.5 | F | n.a. | 3 | - |
| 12 | 28 | F | 4 | 1 | + |
| 13 | 27.5 | F | n.a. | 1 | + |
| 14 | 39 | F | 41 | 1 | + |
| 15 | 27 | M | 21 | 1 | + |
| 16 | 34 | M | n.a. | 1 | - |
| 17 | 51 | M | 20 | 2 |  |
| 18 | 67 | M | n.a. | 3 |  |
| 19 | 46 | F | 25 | 1 |  |
| 20 | 44 | F | 16 | 1 |  |
| 21 | 23 | F | 5 | 2 |  |
| 22 | 53 | M | 10 | 1 |  |
| 23 | 18 | F | 27 | 5 |  |
| 24 | 44 | F | 24 | 2 |  |
| 25 | 64 | F | 330 | 1 |  |
| 26 | 52 | M | 71 | 1 |  |
| 27 | 41 | F | 3960 | 1 |  |

**^a^** time from first symptoms to biopsy

^b^ tissue blocks containing PPWM

+ at least one nodule was associated with APP^+^ axonal profiles

n.a. stands for not available

Supplement 2: 3D image reconstruction showing NPY-Y1R^+^ particles are present within activated microglia/macrophages. Phagocytosed NPY-Y1R^+^ profiles (green) are present in close proximity to the nucleus (blue, counterstained with DAPI) of activated HLA-DR^+^ microglia/macrophages (red); this demonstrates the microglial/macrophage reaction to Wallerian degeneration in early MS non-demyelinated white matter.
